# CdZnS Nanowire Decorated with Graphene for Efficient Photocatalytic Hydrogen Evolution

**DOI:** 10.3390/molecules30143042

**Published:** 2025-07-20

**Authors:** Zemeng Wang, Yunsheng Shen, Qingsheng Liu, Tao Deng, Kangqiang Lu, Zhaoguo Hong

**Affiliations:** 1 Jiangxi Provincial Key Laboratory of Functional Crystalline Materials Chemistry, School of Chemistry and Chemical Engineering, Jiangxi University of Science and Technology, Ganzhou 341000, China; 2 School of Pharmaceutical Sciences, Gannan Medical University, Ganzhou 341000, China

**Keywords:** photocatalysis, hydrogen evolution, cadmium zinc sulfide, cocatalyst, graphene

## Abstract

Harnessing abundant and renewable solar energy for photocatalytic hydrogen production is a highly promising approach to sustainable energy generation. To realize the practical implementation of such systems, the development of photocatalysts that simultaneously exhibit high activity, cost-effectiveness, and long-term stability is critically important. In this study, a Cd_0.8_Zn_0.2_S nanowire photocatalytic system decorated with graphene (GR) was prepared by a simple hydrothermal method. The introduction of graphene increased the reaction active area of Cd_0.8_Zn_0.2_S, promoted the separation of photogenerated charge carriers in the semiconductor, and improved the photocatalytic performance of the Cd_0_._8_Zn_0_._2_S semiconductor. The results showed that Cd_0.8_Zn_0.2_S loaded with 5% graphene exhibited the best photocatalytic activity, with a hydrogen production rate of 1063.4 µmol·g^−1^·h^−1^. Characterization data revealed that the graphene cocatalyst significantly enhances electron transfer kinetics in Cd_0_._8_Zn_0_._2_S, thereby improving the separation efficiency of photogenerated charge carriers. This study demonstrates a rational strategy for designing high-performance, low-cost composite photocatalysts using earth-abundant cocatalysts, advancing sustainable hydrogen production.

## 1. Introduction

The escalating global concerns over climate change, coupled with the ever-increasing energy demand, have catalyzed extensive research into sustainable alternatives to fossil fuels [1,2,3,4]. Given the finite nature of fossil fuel reserves and their detrimental environmental impacts, including greenhouse gas emissions and ecological degradation, there is an urgent imperative to develop and deploy renewable energy technologies as viable substitutes. Hydrogen (H_2_) energy, a clean and renewable fuel, has emerged as one of the most viable alternatives to fossil fuels, offering a sustainable pathway toward decarbonization [5,6,7,8]. Among various methods, photocatalytic hydrogen production driven by solar energy is regarded as a highly promising and sustainable solar energy utilization approach [9,10]. Solar-driven water splitting represents a sustainable and environmentally benign approach to hydrogen production, offering a viable solution to address global energy demands while mitigating environmental challenges associated with fossil fuels [11,12]. Nevertheless, practical applications of semiconductor photocatalysts remain substantially hindered by several intrinsic limitations, including (1) the high recombination rates of photogenerated charge carriers, (2) the escalated costs associated with precious metal cocatalysts, and (3) the unsatisfactory photocatalytic stability under prolonged operation. These critical challenges necessitate the development of economically viable photocatalytic systems with enhanced performance [13,14,15,16,17].

Among various material candidates, metal sulfides have garnered considerable attention in photocatalytic research owing to their unique electronic structures and superior light-harvesting capabilities [18,19]. Notably, cadmium sulfide (CdS) stands out as a particularly promising candidate among metal sulfide photocatalysts due to its (i) exceptional optical absorption properties spanning the UV–visible spectrum, (ii) demonstrated photostability under illumination, and (iii) cost-effectiveness derived from abundant elemental precursors [20,21,22,23]. However, the rapid recombination tendency of photoinduced electrons and holes in single-component cadmium sulfide leads to insufficient active sites, thereby resulting in poor performance. Solid solution metal sulfides are due to their adjustable band gap width and superior charge carrier REDOX capacity. CdS and ZnS can form a solid solution (CdZnS), where the band gap is tunable by varying the stoichiometric ratio of Cd/Zn. This band gap engineering enables optimized light absorption and enhanced photocatalytic water splitting performance. Notably, the rapid recombination of photogenerated electron–hole pairs in CdZnS remains a critical challenge, significantly limiting its photocatalytic efficiency. In addition, the construction of cocatalyzed modified composite materials is expected to enhance photocatalytic activity [24,25]. Graphene, characterized by its remarkable electrical conductivity, demonstrates significant potential to enhance carrier mobility in semiconductors. As an efficient cocatalyst, it not only promotes visible light absorption but also enables efficient carrier migration [26,27,28]. Therefore, the strategy of growing CdZnS nanowires on graphene nanosheets to fabricate graphene/CdZnS composites is highly promising. This approach can effectively improve the separation efficiency of photogenerated electron–hole pairs in CdZnS nanowires, thereby enhancing their photocatalytic performance for water splitting applications.

Herein, a series of GR@Cd_0.8_Zn_0.2_S were prepared using the hydrothermal method. For simplicity, Cd_0.8_Zn_0.2_S is hereinafter referred to as CdZnS. Compared to pure Cd_0.8_Zn_0.2_S (CdZnS), the photocatalytic hydrogen evolution performance of the 5%GR@CdZnS composite catalyst was significantly enhanced. Electrochemical impedance spectroscopy and time-resolved photoluminescence measurements conclusively demonstrated significantly enhanced separation and transfer efficiency of photogenerated charge carriers in the composite catalyst system. This study provides fundamental insights for designing cost-effective composite photocatalysts with superior performance through the strategic incorporation of earth-abundant cocatalysts, offering a sustainable pathway toward scalable solar fuel production.

## 2. Results and Discussion

**Figure 1:**
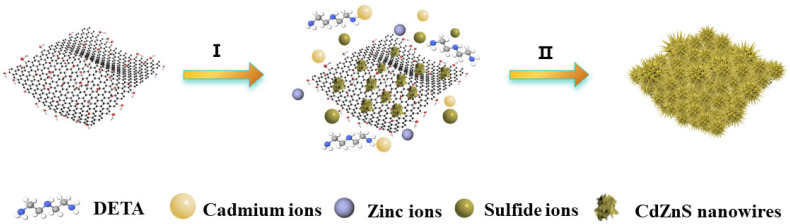
Diagram illustrating the synthesis of GR@CdZnS.

The preparation process of GR/CdZnS composite materials is illustrated in Figure 1. GR/CdZnS composite materials were prepared using a one-step hydrothermal method at a high temperature. In a stainless-steel autoclave lined with Teflon, Diethylenetriamine (DETA) molecules first protonate and react with water at 160 °C, forming positively charged ammonium ions. Subsequently, the protonated DETA molecules coordinate with sulfur atoms in adjacent CdZnS layers, modifying the DETA molecules to construct the CdZnS nanowires. Finally, the surface of GR is modified with CdZnS nanowires to form the GR@CdZnS composite material through a solvent-thermal reaction.

**Figure 2:**
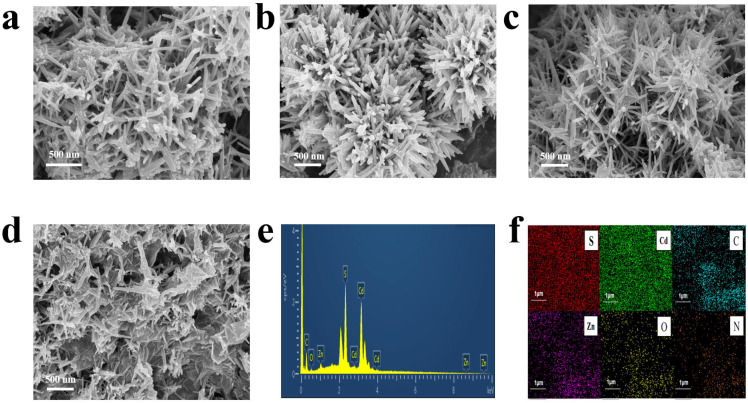
(**a**,**b**) SEM images of CdZnS. (**c**,**d**) SEM images of GR@CdZnS. (**e**) EDS of GR@CdZnS. (**f**) Mapping analysis results of GR@CdZnS.

Scanning electron microscopy (SEM) was employed to characterize the morphological features and microstructural characteristics of various samples. As revealed in Figure 2a,b, the pure CdZnS sample consists of spiky microspheres with an average diameter of 1 μm, as determined by SEM analysis. As shown in Figure 2c, the SEM image of GR@CdZnS reveals a well-preserved spiky spherical morphology, consistent with the pure CdZnS structure [29]. Notably, the spike-like structures on the surface of the spheres increase the catalyst’s contact surface area, providing a larger specific surface area, enhancing light absorption by building on the already substantial surface area of the spherical structure, and offering more active sites [30]. Additionally, the SEM image of GR@CdZnS reveals highly dispersed CdZnS nanoparticles without the presence of large particles (Figure 2d). Further SEM characterization (Figure 2d) confirms the spiky spherical structure of CdZnS. Furthermore, energy-dispersive X-ray spectroscopy (EDS) spectra (Figure 2e) and elemental mapping images (Figure 2f) provide evidence for the coexistence of Zn, N, S, Cd, O, and C in GR@CdZnS. The spatial distribution patterns of Zn, N, S, Cd, O, and C revealed by the elemental mapping of the GR@CdZnS composite materials suggest the homogeneous growth of CdZnS onto the graphene surface.

**Figure 3:**
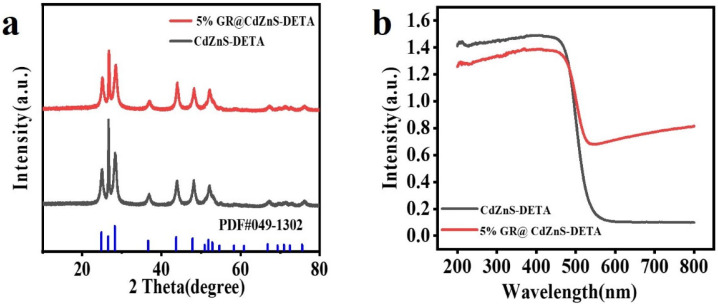
(**a**) X-ray diffraction (XRD) patterns and (**b**) UV–vis diffuse reflectance spectra (DRS) of CdZnS and GR@CdZnS.

The phase structures and crystallinity of the materials were analyzed using X-ray diffraction (XRD) patterns. Figure 3a presents the XRD spectra of CdZnS and GR@CdZnS. For CdZnS, the strong diffraction peaks at 25.4°, 27.2°, 28.9°, 45.1°, 49.2°, and 53.5° correspond to the (100), (002), (101), (110), (103), and (112) planes of CdZnS (JCPDS card No. 49-1302) [3,31]. For the GR@CdZnS composite material, its XRD pattern exhibits a close similarity to that of CdZnS. No graphene peaks were observed in the spectrum, which can be attributed to the low content of graphene in the composite material and the relatively weak crystalline phase structure of graphene. The optical properties of the photocatalysts were investigated via ultraviolet–visible diffuse reflectance spectroscopy (DRS). As depicted in Figure 3b, pure CdZnS displays a distinct absorption edge at approximately 542 nm. In comparison to pure CdZnS, the GR@CdZnS composite materials exhibit enhanced absorption intensity in the visible light region (560–780 nm) due to the strong light-harvesting capacity of GR. The light absorption edge of the composite is observed at approximately 580 nm, suggesting that the incorporation of GR improves the visible light responsiveness of CdZnS.

**Figure 4:**
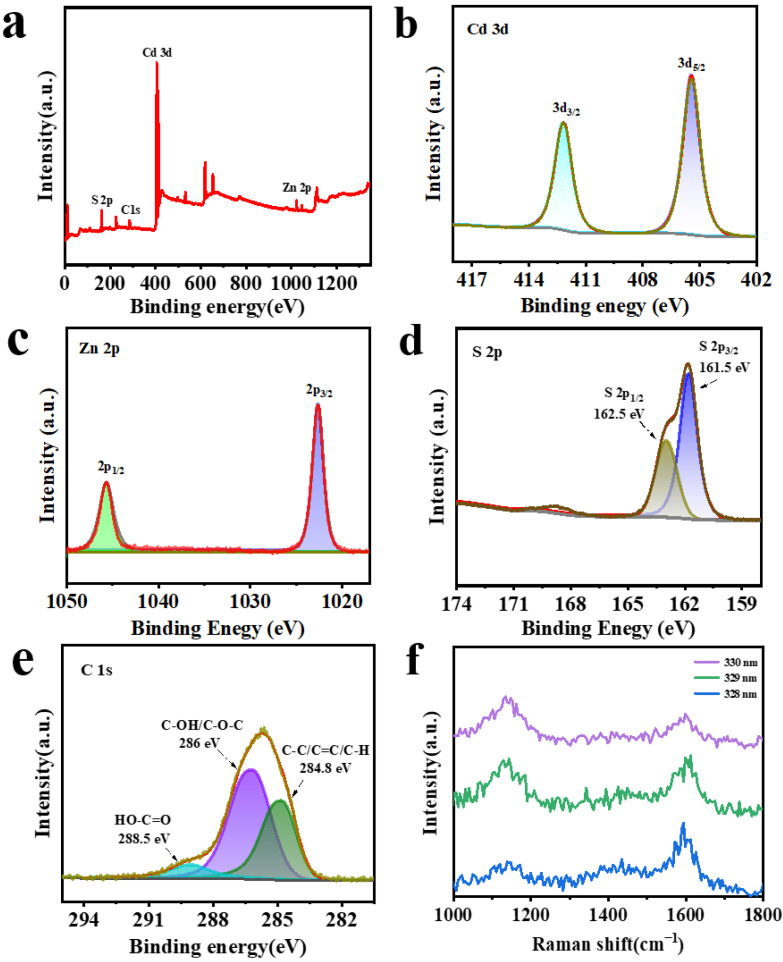
(**a**) XPS spectra of GR@CdZnS, high-resolution spectra of (**b**) Cd 3d, (**c**) Zn 2p, (**d**) S 2p, (**e**) C 1s, (**f**) Raman spectra of GR@CdZnS.

X-ray photoelectron spectroscopy (XPS) was employed to further characterize the chemical composition and elemental valence states of the GR@CdZnS composite material. As shown in Figure 4a, the hybrid product contains the elements Zn, S, C, and Cd. The XPS spectrum of Cd 3d (Figure 4b) displays two peaks at binding energies of 412.4 eV and 405.8 eV, which are assigned to Cd 3d_3_/_2_ and Cd 3d_5_/_2_, respectively [32]. The XPS spectrum of Zn^2+^ in Figure 4c exhibits two distinct peaks at 1045 eV and 1022 eV, corresponding to the Zn 2p_1/2_ and Zn 2p_3/2_ binding energies, respectively [33]. Furthermore, the peaks at 161.5 eV and 162.5 eV in Figure 4d can be assigned to S 2p_3_/_2_ and S 2p_1_/_2_, providing evidence for the existence of S^2−^ in CdZnS [34]. The C 1s XPS spectrum (Figure 4e) of the GR@CdZnS hybrid material demonstrates significant reductions in oxygen-containing functional groups, verifying the efficient conversion of graphene oxide to graphene through the thermal reflux process [35]. Additionally, Raman spectroscopy analysis, as shown in Figure 4f, reveals that the peaks at 1153 and 1570 cm^−1^ can be assigned to the D band and G band of graphene, respectively [36,37].

**Figure 5:**
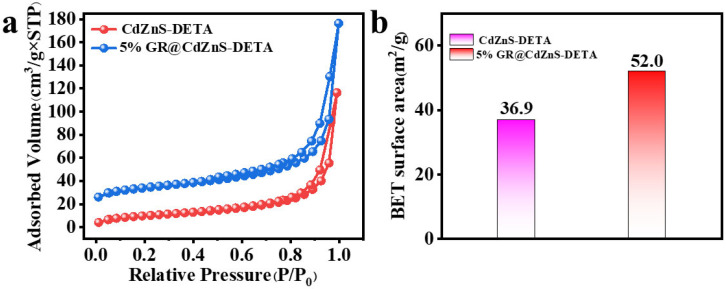
(**a**) Isotherms for N_2_ adsorption–desorption of CdZnS and GR@CdZnS and (**b**) BET surface area versus CdZnS and GR@CdZnS.

Figure 5a and Figure S1 shows the N_2_ gas adsorption–desorption isotherms for CdZnS, GR/CdZnS hybrids and GR. The BET surface area values (SBET) are summarized in Figure 5b and Table S1. It is found that GR/CdZnS hybrids (52.0 m^2^/g) have a larger surface area than that of pure CdZnS (36.9 m^2^/g). The results indicate that GR/CdZnS hybrids with larger SBET can provide more active sites, which is also a positive factor for higher photocatalytic hydrogen evolution performance.

**Figure 6:**
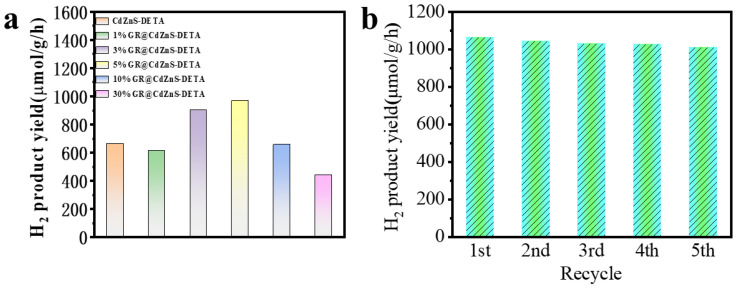
(**a**) Photocatalytic H_2_ production over pure CdZnS and GR@CdZnS composites. (**b**) Stability plots of the photocatalytic H_2_ production by GR@CdZnS.

Utilizing triethanolamine (TEOA) as a sacrificial agent for photocatalytic hydrogen production, the study investigated the visible light photocatalytic performance of pure CdZnS and CdZnS/GR composite materials with varying ratios. Figure 6a illustrates the photocatalytic activity of CdZnS and composite materials under conditions of 1%, 3%, 5%, 10%, and 30% GR. As depicted in Figure 6a, due to the rapid recombination rate of photogenerated electrons and holes, pure CdZnS exhibits low activity with a hydrogen evolution rate of only 662.9 μmol∙g^−1^∙h^−1^. Upon introducing GR as a cocatalyst, the hydrogen evolution performance of 3% and 5% GR surpasses that of pure CdZnS. Notably, the 5% GR@CdZnS composite material achieves a maximum hydrogen evolution rate of 1063.4 μmol·g^−1^·h^−1^, which is approximately 1.5-fold higher than that of pure CdZnS. This improvement is ascribable to the superior electrical conductivity of GR and high charge carrier mobility, which promote the migration of photogenerated electrons and consequently decrease the recombination rate of charge carriers. Despite their promising photocatalytic properties, transition metal sulfides often suffer from severe photocorrosion, leading to rapid performance degradation and limited operational lifetimes. These intrinsic stability issues pose significant challenges to their long-term viability and industrial-scale implementation. As demonstrated in Figure 6b, the 5% GR@CdZnS composite exhibits exceptional stability, with no significant activity loss observed after five consecutive photocatalytic cycles. This remarkable durability highlights the effectiveness of the GR modification strategy in suppressing photocorrosion and enhancing practical applicability.

**Figure 7:**
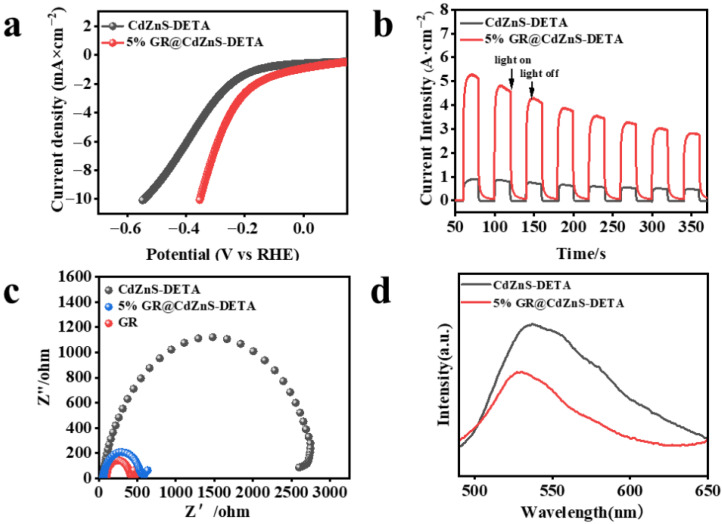
(**a**) Polarization curves of CdZnS and GR@CdZnS composite. (**b**) Photocurrent–time (I-t) curves of CdZnS and GR@CdZnS composite. (**c**) Nyquist plots of EIS measurements on GR, CdZnS, and GR@CdZnS composite. (**d**) Photoluminescence (PL) spectra of CdZnS and GR@CdZnS composite.

The hydrogen evolution performance of CdZnS and GR@CdZnS samples was evaluated using linear sweep voltammetry (LSV). Figure 7a illustrates the polarization curves for CdZnS and GR@CdZnS composite materials. It is observed that at the same current density, the overpotential of GR@CdZnS is lower than that of CdZnS, indicating superior hydrogen evolution performance of GR@CdZnS compared to CdZnS [38]. Electrochemical and photoelectrochemical tests were conducted to further characterize the reduction capability of materials and the efficiency of carrier transfer generated by light. Instantaneous photocurrent (IT) and electrochemical impedance spectroscopy (EIS) measurements were conducted on CdZnS and 5% GR@CdZnS samples to investigate charge separation and transfer processes in these composite materials. The photocurrent density of CdZnS, as shown in Figure 7b, was relatively low, indicating the poor separation efficiency of photogenerated carriers. However, with the introduction of graphene (GR), the photocurrent density of 5%GR@CdZnS significantly increased compared to pure CdZnS, indicating enhanced electron (e^−^) and hole (h^+^) separation efficiency [39]. Figure 7c demonstrates that the curvature radius of the 5% GR@CdZnS composite material is smaller than that of CdZnS, indicating reduced charge transfer resistance in the 5% GR@CdZnS system. This reduction facilitates the more efficient separation and transfer of photogenerated carriers, thereby enhancing the photocatalytic activity [40]. As illustrated in Figure 7d, the photoluminescence (PL) intensity of the 5% GR@CdZnS composite is markedly lower than that of pure CdZnS. Catalyst systems incorporating cocatalysts display substantially reduced PL intensity and shorter PL lifetimes compared to the blank system. Typically, diminished PL emission intensity and abbreviated PL lifetimes signify the more efficient suppression of photoexcited charge carrier recombination, implying that the introduction of the cocatalyst GR effectively hinders the recombination of photogenerated charge carriers [41].

**Figure 8:**
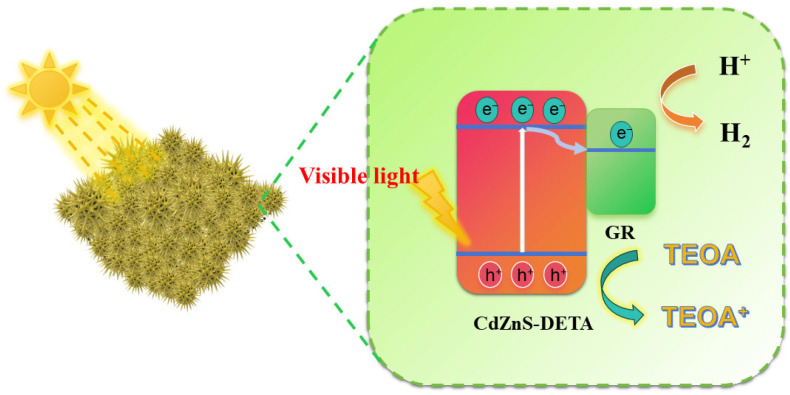
Mechanism diagram of GR@CdZnS in the visible light-driven photocatalytic H_2_ production reaction.

On the basis of the aforementioned experiments and characterizations, a plausible mechanism for visible light-driven hydrogen production over the GR@CdZnS photocatalyst is proposed. As illustrated in Figure 8, upon visible light absorption, electrons in CdZnS are excited and migrate to its conduction band, generating holes in the CdZnS valence band. Subsequently, these photoexcited electrons then transfer to the GR sites, where they combine with protons from water to produce H_2_. Meanwhile, triethanolamine (TEOA) is oxidized by the accumulated holes in CdZnS’s valence band to form oxidized triethanolamine. The introduction of a small amount of GR significantly enhances electron–hole pair separation and provides abundant active sites, thereby improving the photocatalytic H_2_ production performance of GR@CdZnS composites.

## 3. Experimental Section

### 3.1. Materials

Graphite powder, concentrated sulfuric acid (H_2_SO_4_), potassium permanganate (KMnO_4_), hydrogen peroxide (H_2_O_2_), 1:10 dilute hydrochloric acid, cadmium nitrate tetrahydrate (Cd(NO_3_)_2_·4H_2_O), zinc nitrate hexahydrate (Zn(NO_3_)_2_·6H_2_O), thiourea (CH_4_N_2_S), diethylenetriamine (C_4_H_1__3_N_3_), and ethanol (C_2_H_5_OH) were provided by Sinopharm Chemical Reagent Co., Ltd. (Shanghai, China). In the experiments, the chemicals used were all of analytical grade. The deionized water (DI) used in the experiments was sourced from local laboratories.

### 3.2. Synthesis of GR@CdZnS

To begin, 51.0 mg of Cd(NO_3_)_2_·4H_2_O, 12.3 mg of Zn(NO_3_)_2_·6H_2_O, and 15.7 mg of thiourea were added to a mixed solution of 14.4 mL of H_2_O and 3.6 mL of DETA and stirred for 5 min. Then, 1.25 mg of graphene oxide (GO) was added to the above solution and stirred for 30 min [42]. Afterwards, the homogeneous mixture was transferred into a 50 mL Teflon-lined stainless-steel autoclave and hydrothermally treated at 160 °C for 8 h. During this process, the heat treatment induces the reduction of GO, eventually forming reduced graphene oxide, hereafter denoted as graphene (GR). The subsequent C 1s XPS spectrum and the Raman spectroscopy results provide robust evidence for this assertion. After cooling to room temperature, the resulting products were sequentially washed with absolute ethanol and deionized water (3× each) to remove impurities, followed by vacuum drying to yield the final 5%-GR@CdZnS composite. By adjusting the feed amounts of GO but keeping the addition of Cd(NO_3_)_2_·4H_2_O, Zn(NO_3_)_2_·6H_2_O, and thiourea unchanged, other samples were achieved. The GR sample was prepared by subjecting 1.25 mg of GO to the same treatment protocol described above, with the exception that CdZnS precursors, including Cd(NO_3_)_2_·4H_2_O, Zn(NO_3_)_2_·6H_2_O, and thiourea, were not added.

### 3.3. Photocatalytic Activity Evaluation H_2_ Evolution

The photocatalytic hydrogen evolution experiments were conducted in a 50 mL airtight quartz reactor under ambient conditions. A photocatalyst suspension was prepared by dispersing 5 mg of catalyst in a 6 mL aqueous solution containing 5 mL of deionized water and 1 mL of triethanolamine (TEOA) as a sacrificial agent. Prior to illumination, the reactor was purged with high-purity argon for 30 min to exhaust the residual air in the reactor. A 300 W xenon lamp (PLS-SXE300D, Perfectlight, Beijing, China) with a wavelength longer than 420 nm was utilized as the light-emitting source. Following a 2 h illumination period, 1 mL of gas was withdrawn and introduced into a gas chromatograph fitted with a thermal conductivity detector (TCD) for the purpose of quantifying the hydrogen generation yield resulting from the reaction.

## 4. Conclusions

In conclusion, a series of CdZnS/GR composite photocatalysts were successfully synthesized via a facile hydrothermal approach. Compared with pure CdZnS, the binary CdZnS/GR composite demonstrated a significantly enhanced performance. The optimal photocatalytic hydrogen evolution rate of CdZnS/5% GR reached 1063.4 μmol∙g^−1^∙h^−1^, which was 1.5 times higher than that of pure CdZnS. The comprehensive characterization findings suggest that the introduction of the cocatalyst GR offers extra active sites for H_2_ generation. It efficiently accelerates the separation of photogenerated electron–hole pairs in CdZnS, diminishes the recombination rate, and boosts the photocatalytic hydrogen evolution efficiency. This research is expected to provide valuable perspectives on the rational design of photocatalysts for hydrogen production and the development of high-performance, selective composite photocatalysts.

## Data Availability

Data are contained within the article and Supplementary Materials.

## Supplementary Materials

The following supporting information can be downloaded at https://www.mdpi.com/article/10.3390/molecules30143042/s1, Figure S1: Isotherms for N_2_ adsorption-desorption of GR; Table S1: BET surface area of GR, CdZnS and 5%GR@CdZnS.

## References

[1] Shi Z., Jin W., Sun Y.H., Li X., Mao L., Cai X.Y., Lou Z.Z. (2023). Interface charge separation in Cu_2_CoSnS_4_/ZnIn_2_S_4_ heterojunction for boosting photocatalytic hydrogen production. Chin. J. Struct. Chem..

[2] He H., Wang Z., Zhang J., Shao C., Dai K., Fan K. (2024). Interface Chemical Bond Enhanced Ions Intercalated Carbon Nitride/CdSe-Diethylenetriamine S-Scheme Heterojunction for Photocatalytic H_2_O_2_ Synthesis in Pure Water. Adv. Funct. Mater..

[3] Wang X., Liu B.Y., Ma S.Q., Zhang Y.J., Wang L.Z., Zhu G.Q., Huang W., Wang S.C. (2024). Induced dipole moments in amorphous ZnCdS catalysts facilitate photocatalytic H_2_ evolution. Nat. Commun..

[4] Zheng M., Wu P., Li L., Yu F., Ma J. (2023). Adsorption/desorption behavior of ciprofloxacin on aged biodegradable plastic PLA under different exposure conditions. J. Environ. Chem. Eng..

[5] Zhang J.H., Wang Y.C., Wang H.J., Zhong D.C., Lu T.B. (2022). Enhancing photocatalytic performance of metal-organic frameworks for CO_2_ reduction by a bimetallic strategy. Chin. Chem. Lett..

[6] Wu Y.-H., Yan Y.-Q., Wei Y., Wang J., Li A., Huang W.-Y., Zhang J.-L., Yang K., Lu K.-Q. (2024). Decorating ZnIn_2_S_4_ with earth-abundant Co_9_S_8_ and Ni_2_P dual cocatalysts for boosting photocatalytic hydrogen evolution. Int. J. Hydrogen Energy.

[7] Li M., Van Der Veer M., Yang X., Weng B., Shen L., Huang H., Dong X., Wang G., Roeffaers M.B.J., Yang M.-Q. (2024). Twin boundary defect engineering in Au cocatalyst to promote alcohol splitting for coproduction of H_2_ and fine chemicals. J. Colloid Interface Sci..

[8] Pan Z.M., Zhang G.G., Wang X.C. (2019). Polymeric Carbon Nitride/Reduced Graphene Oxide/Fe_2_O_3_: All-Solid-State Z-Scheme System for Photocatalytic Overall Water Splitting. Angew. Chem.-Int. Ed..

[9] Jin Y.X., Zheng D.D., Fang Z.P., Pan Z.M., Wang S.B., Hou Y.D., Savateev O., Zhang Y.F., Zhang G.G. (2024). Salt-melt synthesis of poly(heptazine imide) in binary alkali metal bromides for enhanced visible-light photocatalytic hydrogen production. Interdiscip. Mater..

[10] Weng Z., Lin Y., Guo S., Zhang X., Guo Q., Luo Y., Ou X., Ma J., Zhou Y., Jiang J. (2023). Site Engineering of Covalent Organic Frameworks for Regulating Peroxymonosulfate Activation to Generate Singlet Oxygen with 100 % Selectivity. Angew. Chem. Int. Ed..

[11] Fu W., Fan J.J., Xiang Q.J. (2022). Ag_2_S quantum dots decorated on porous cubic-CdS nanosheets-assembled flowers for photocatalytic CO_2_ Reduction. Chin. J. Struct. Chem..

[12] Zheng X.L., Yang Y.Q., Song Y.M., Ma Z.X., Gao Q.Z., Liu Y.H., Li J., Wu X., Wang X.B., Mao W.H. (2023). Recent advances in photocatalytic hydrogen evolution of AgIn_5_S_8_-based photocatalysts. Interdiscip. Mater..

[13] Yan Y.Q., Wu Y.Z., Wu Y.H., Weng Z.L., Liu S.J., Liu Z.G., Lu K.Q., Han B. (2024). Recent advances of CeO_2_-based composite materials for photocatalytic applications. ChemSusChem.

[14] Liu X., Zhang Y., Wang C., Shen L. (2024). Polar materials for photocatalytic applications: A critical review. Interdiscip. Mater..

[15] Liu L., Wang Z., Zhang J., Ruzimuradov O., Dai K., Low J. (2023). Tunable Interfacial Charge Transfer in a 2D–2D Composite for Efficient Visible-Light-Driven CO_2_ Conversion. Adv. Mater..

[16] Li S., Dong K., Cai M., Li X., Chen X. (2024). A plasmonic S-scheme Au/MIL-101(Fe)/BiOBr photocatalyst for efficient synchronous decontamination of Cr(VI) and norfloxacin antibiotic. eScience.

[17] Su B., Kong Y., Wang S., Zuo S., Lin W., Fang Y., Hou Y., Zhang G., Zhang H., Wang X. (2023). Hydroxyl-Bonded Ru on Metallic TiN Surface Catalyzing CO_2_ Reduction with H_2_O by Infrared Light. J. Am. Chem. Soc..

[18] Wei Y., Wu Y., Wang J., Wu Y.-H., Weng Z., Huang W.-Y., Yang K., Zhang J.-L., Li Q., Lu K.-Q. (2024). Rationally designed dual cocatalysts on ZnIn_2_S_4_ nanoflowers for photoredox coupling of benzyl alcohol oxidation with H_2_ evolution. J. Mater. Chem. A.

[19] Weng Z., Lin Y., Han B., Zhang X., Guo Q., Luo Y., Ou X., Zhou Y., Jiang J. (2023). Donor-acceptor engineered g-C_3_N_4_ enabling peroxymonosulfate photocatalytic conversion to ^1^O_2_ with nearly 100% selectivity. J. Hazard. Mater..

[20] Jing D.W., Guo L.J. (2006). A novel method for the preparation of a highly stable and active CdS photocatalyst with a special surface nanostructure. J. Phys. Chem. B.

[21] Hu Y., Gao X.H., Yu L., Wang Y.R., Ning J.Q., Xu S.J., Lou X.W. (2013). Carbon-Coated CdS Petalous Nanostructures with Enhanced Photostability and Photocatalytic Activity. Angew. Chem.-Int. Ed..

[22] Ke D.N., Liu S.L., Dai K., Zhou J.P., Zhang L., Peng T.Y. (2009). CdS/regenerated cellulose nanocomposite films for highly efficient photocatalytic H_2_ production under visible light irradiation. J. Phys. Chem. C.

[23] Kudo A., Miseki Y. (2009). Heterogeneous photocatalyst materials for water splitting. Chem. Soc. Rev..

[24] Zou J., Wu S., Liu Y., Sun Y., Cao Y., Hsu J.-P., Shen Wee A.T., Jiang J. (2018). An ultra-sensitive electrochemical sensor based on 2D g-C_3_N_4_/CuO nanocomposites for dopamine detection. Carbon.

[25] Cai M., Liu Y., Dong K., Chen X., Li S. (2023). Floatable S-scheme Bi_2_WO_6_/C_3_N_4_/carbon fiber cloth composite photocatalyst for efficient water decontamination. Chin. J. Catal..

[26] Chen H., Zhou Y.S., Guo W., Xia B.Y. (2022). Emerging two-dimensional nanocatalysts for electrocatalytic hydrogen production. Chin. Chem. Lett..

[27] Lu K.-Q., Hao J.-G., Wei Y., Weng B., Ge S., Yang K., Lu S., Yang M.-Q., Liao Y. (2023). Photocatalytic conversion of diluted CO_2_ into tunable syngas via modulating transition metal hydroxides. Inorg. Chem..

[28] Lu K.-Q., Li Y.-H., Zhang F., Qi M.-Y., Chen X., Tang Z.-R., Yamada Y.M.A., Anpo M., Conte M., Xu Y.-J. (2020). Rationally designed transition metal hydroxide nanosheet arrays on graphene for artificial CO_2_ reduction. Nat. Commun..

[29] Wang J., Li B., Chen J., Li L., Zhao J., Zhu Z. (2013). Hierarchical assemblies of Cd_x_Zn_1−x_S complex architectures and their enhanced visible-light photocatalytic activities for H_2_-production. J. Alloys Compd..

[30] Zhou Y., Wang Y., Wen T., Zhang S., Chang B., Guo Y., Yang B. (2016). Mesoporous Cd_1−x_Zn_x_S microspheres with tunable bandgap and high specific surface areas for enhanced visible-light-driven hydrogen generation. J. Colloid Interface Sci..

[31] Xing C.J., Zhang Y.J., Yan W., Guo L.J. (2006). And structure-controlled solid solution of Cd_1-x_Zn_x_S photocatalyst for hydrogen production by water splitting. Int. J. Hydrogen Energy.

[32] Wang G.R., Quan Y.K., Yang K.C., Jin Z.L. (2022). EDA-assisted synthesis of multifunctional snowflake-Cu_2_S/CdZnS S-scheme heterojunction for improved the photocatalytic hydrogen evolution. J. Mater. Sci. Technol..

[33] Li H., Tao S.R., Wan S.J., Qiu G.G., Long Q., Yu J.G., Cao S.W. (2023). S-scheme heterojunction of ZnCdS nanospheres and dibenzothiophene modified graphite carbon nitride for enhanced H_2_ production. Chin. J. Catal..

[34] Xue W.H., Chang W.X., Hu X.Y., Fan J., Liu E.Z. (2021). 2D mesoporous ultrathin Cd_0.5_Zn_0.5_S nanosheet: Fabrication mechanism and application potential for photocatalytic H_2_ evolution. Chin. J. Catal..

[35] Liu Z.-G., Wei Y., Xie L., Chen H.-Q., Wang J., Yang K., Zou L.-X., Deng T., Lu K.-Q. (2024). Decorating CdS with cobaltous hydroxide and graphene dual cocatalyst for photocatalytic hydrogen production coupled selective benzyl alcohol oxidation. Mol. Catal..

[36] Jiang J., Ou-yang L., Zhu L., Zheng A., Zou J., Yi X., Tang H. (2014). Dependence of electronic structure of g-C _3_N_4_ on the layer number of its nanosheets: A study by Raman spectroscopy coupled with first-principles calculations. Carbon.

[37] Tang Z.L., He W.J., Wang Y.L., Wei Y.C., Yu X.L., Xiong J., Wang X., Zhang X., Zhao Z., Liu J. (2022). Ternary heterojunction in rGO-coated Ag/Cu_2_O catalysts for boosting selective photocatalytic CO_2_ reduction into CH_4_. Appl. Catal. B-Environ. Energy.

[38] Wu Y.L., Li Y.J., Zhang L.J., Jin Z.L. (2022). NiAl-LDH In-Situ Derived Ni_2_P and ZnCdS Nanoparticles Ingeniously Constructed S-Scheme Heterojunction for Photocatalytic Hydrogen Evolution. ChemCatChem.

[39] Tian J.Z., Cao X.F., Sun T., Fan J., Miao H., Chen Z., Li D., Liu E.Z., Zhu Y.H. (2023). S-scheme Co_3_(PO_4_)_2_/Twinned-Cd_0.5_Zn_0.5_S homo-heterojunction for enhanced photocatalytic H_2_ evolution. Chem. Eng. J..

[40] He K., Guo L.J. (2023). ZnS/Cd_1-x_Zn_x_S composite photocatalyst synthesized with excessive strong alkali solution as a hydrothermal solvent: The chemical equilibrium theory of the synthesis process and its influence on photocatalytic performance. Int. J. Hydrogen Energy.

[41] Wang X.W., Wang W.Y., Du B., Zhou C.X., Feng G., Cai J.X., Wang T., Zhang R.B. (2017). Noble metal-free Cd_1-x_Zn_x_S-Zn_1-y_Cd_y_S heterostructures for stable and highly effective photocatalytic hydrogen evolution. J. Alloys Compd..

[42] Su B., Zheng M., Lin W., Lu X.F., Luan D., Wang S., Lou X.W. (2023). S-scheme Co_9_S_8_@Cd_0.8_Zn_0.2_S-DETA hierarchical nanocages bearing organic CO_2_ activators for photocatalytic syngas production. Adv. Energy Mater..

