# Supplementary material for: CdZnS Nanowire Decorated with Graphene for Efficient Photocatalytic Hydrogen Evolution
Source: Molecules. 2025 Jul 20;30(14):3042. doi: 10.3390/molecules30143042 (PMC12299082; doi:10.3390/molecules30143042)
Supplement: Supplementary file 1 [file molecules-30-03042-s001.zip › molecules-3721428-supplementary.pdf]

Supporting information

CdZnS Nanowire Decorated with Graphene for Efficient Photocatalytic Hydrogen Evolution

Zemeng Wang [1], Yunsheng Shen [1], Qingsheng Liu [1], Tao Deng [1], Kangqiang Lu [1,*] and Zhaoguo Hong [2,*]

[1] Jiangxi Provincial Key Laboratory of Functional Crystalline Materials Chemistry, School of Chemistry and Chemical Engineering, Jiangxi University of Science and Technology, Ganzhou 341000, China; mmeng8686@163.com (Z.W.); shenyunsheng1@163.com (Y.S.); 1320221607@mail.jxust.edu.cn (Q.L.); taodeng123@163.com (T.D.)

[2] School of Pharmaceutical Sciences, Gannan Medical University, Ganzhou 341000, China

* **Correspondence:** kqlu@jxust.edu.cn (K.L.); 18677342951@163.com (Z.H.)

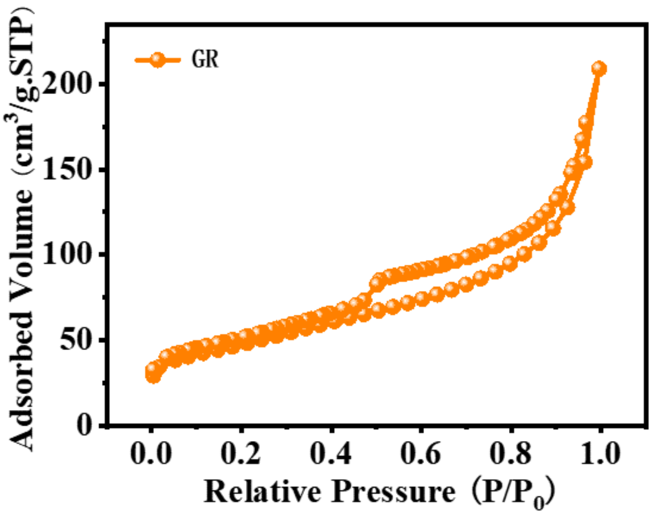

Supplementary Figure S1. Isotherms for N_2 adsorption–desorption of GR.

Supplementary Table S1. BET surface area of GR, CdZnS, and 5%GR@CdZnS.

Samples	BET surface area ($m^2\ g^{-1}$)
GR	295
CdZnS	36.9
5%GR@CdZnS	52.0

Synthetic procedures and characterization of products

1：Preparation of Graphene Oxide

Graphene oxide solution (GO) was prepared using a modified Hummers' method. Under moderate stirring, 10 g of graphite powder was added to 230 mL of concentrated H_2SO_4. While stirring continuously, 30 g of $KMnO_4$ was gradually introduced, and the solution was cooled to below 5 °C in an ice bath. The mixture was then heated to 35 °C in a water bath and magnetically stirred for 2 hours. Subsequently, the mixture was slowly diluted with 500 mL of deionized water in an ice bath to maintain the temperature below 5 °C, followed by further dilution with 1500 mL of deionized water. Then, 80 mL of 30% H_2O_2 was slowly added to the mixture. The resulting mixture was centrifuged and washed with a 1:10 (concentrated HCl/deionized water) aqueous HCl solution to remove metal ions, followed by washing with deionized water to remove acid residues. Finally, the mixture was dialyzed against deionized water for one week, with the deionized water replaced every 12 hours, yielding a graphene oxide solution after dialysis.

2：Characterization Methods

The crystal phase structure and composition of the catalyst were determined by X-ray diffraction (Bruker D8 Advance, Bruker Corporation, Saarbrucken, Germany). A scanning electron microscope (FESEM Zeiss Sigma 500, Zeiss, Oberkochen, Germany) was used to analyze the morphology and microstructure of the composite photocatalyst. The composition and valence of the composite photocatalyst were analyzed by Thermo Fisher K-Alpha Plus (X-ray photoelectron spectroscopy) (Thermo Fisher, Waltham, America). In H_2 evolution performance evaluation, the 300 W xenon lamp (PLS-SXE300D, Perfectlight, Beijing, China) and the gas chromatograph (GC7900, Techcomp, Shanghai, China) were employed. Photoluminescence (PL) spectra were obtained using a spectrofluorometer (FLS 980, Edinburgh Instruments Ltd., Edinburgh, UK) with an excitation wavelength of 500 nm. Furthermore, all the electrochemical measurements of the photocurrent and the electrochemical impedance spectra (EIS) were carried out in the three-electrode cell, in which Ag/AgCl was used as a reference electrode, a Pt wire was used as a counter electrode, and a fluorine-doped tin oxide (FTO) conductive glass was used with the samples as a working electrode in 0.1 M Na_2SO_4 electrolyte (pH = 7.56). The working electrode was fabricated on FTO glass, which was cleaned by ultrasonication in ethanol for 30 minutes and subsequently dried at 80 °C. Typically, 3 mg of the sample powder was ultrasonicated in 0.5 mL of DMF

to achieve uniform dispersion, forming a slurry. This slurry was then spread onto the FTO glass, whose edge regions had been pre-protected with Scotch tape. Following air drying, the working electrode was further dried at 100 °C for 2 hours to enhance adhesion. After removing the Scotch tape, the uncoated areas of the electrode were insulated with epoxy resin, resulting in an exposed working area of 0.25 cm^2. All measurements were carried out on CH Instruments' CHI-660E electrochemical workstation (Shanghai Chenhua CHI-660E, Shanghai, China). The specific surface area and pore size of the composite photocatalyst were determined by nitrogen physical adsorption and desorption (ASAP2020). The UV–visible diffuse reflectance spectrometer (DRS, Shimadzu UV-2600, Kyoto, Japan) was utilized to test the optical response of the catalyst.
